# Perceived Discrimination and Cognitive Function Among Older Adults: An Analysis of the Health and Retirement Study

**DOI:** 10.1007/s40615-026-03174-w

**Published:** 2026-09-17

**Authors:** Monique S. Balthazar, Galappaththige de Silva, Karolus Wangi, Donna Fick, Sunday Azagba

**Affiliations:** 1 Ross and Carol Nese College of Nursing, Pennsylvania State University, PAUniversity Park 16802, USA

**Keywords:** Perceived discrimination, cognitive function, older adults

## Abstract

**Background:**

Cognitive aging is a major public health concern, and cognitive health disparities disproportionately affect racially and ethnically minoritized communities. Perceived everyday discrimination has been proposed as a contributor to disparities in cognitive function. This study examines associations between perceived everyday discrimination and cognitive function among older U.S. adults and describes patterns across racial and ethnic groups.

**Methods:**

Data from the Health and Retirement Study (2008–2022) included 45,089 observations from 20,020 participants (aged ≥ 50). Cognitive function was assessed via the adapted Telephone Interview for Cognitive Status, yielding global cognition and episodic memory scores. Perceived everyday discrimination was measured using the 6-item Everyday Discrimination Scale. Associations were evaluated using weighted generalized estimating equations, adjusting for sociodemographic characteristics, educational attainment, health indicators, and survey wave. Analyses incorporated the HRS sampling design, survey weights, longitudinal inverse probability weights for attrition and Leave-Behind nonresponse, and repeated observations within participants.

**Results:**

Non-Hispanic (NH) Black participants reported the highest mean perceived discrimination scores (0.75), followed by NH Other (0.74), whereas NH White (0.53) and Hispanic (0.58) participants reported lower scores. In the full sample, higher perceived discrimination was associated with lower global cognition (estimate: −0.43; 95% CI: −0.52, −0.33) and lower episodic memory (−0.30; 95% CI: −0.37, −0.24). In race- and ethnicity-stratified analyses, inverse associations between perceived discrimination and global cognition were observed among Hispanic, NH Black, and NH White participants. Among these groups, estimates ranged from −0.47 among Hispanic participants (95% CI: −0.66, −0.28) to −0.42 among NH White participants (95% CI: −0.53, −0.31).

**Conclusions:**

Perceived discrimination is associated with lower cognitive performance. Although exposure levels vary across racial and ethnic groups, associations with cognition are broadly consistent in direction, highlighting perceived discrimination as an important social correlate of cognitive health among aging populations.

## Introduction

Maintaining cognitive health in later life is a growing public health priority as the United States (U.S.) population ages [1, 2]. Age-related declines in memory, processing speed, and executive function vary considerably, and poorer cognitive performance has been associated with reduced independence, diminished well-being, and elevated dementia risk [3, 4]. Identifying modifiable social determinants that contribute to this variation is therefore increasingly important.

Racial and ethnic disparities in cognitive function among older adults are well documented [5, 6], with economically disadvantaged, rural, and racially minoritized communities experiencing lower average cognitive performance – populations with a greater likelihood of exposure to adverse living, learning, and working conditions [7]. The association between socioeconomic status (SES) and cognitive function in the U.S. is due in part to systemic inequality, resulting in differential access [8, 9] to quality education and other resources, based largely on race taxonomy [5, 10–14]. Consequently, Black American individuals, who constitute approximately 13% of the U.S. population [15], tend to demonstrate lower mean scores on standardized cognitive assessments compared to Non-Hispanic (NH) White American individuals, disparities that are not fully explained by SES [5, 6]. A growing body of extant literature suggests that the chronic stress associated with experiences of everyday discrimination may be a significant contributing factor [6, 16, 17].

Perceived everyday discrimination — defined here as the frequency of routine experiences of unfair treatment as captured by the Everyday Discrimination Scale [18] — is a chronic stressor with potentially detrimental effects on cognitive function [19, 20]. Recent work using data from the Health and Retirement Study found that chronic stress and perceived discrimination together accounted for approximately one-quarter of the observed Black-White disparity in incident dementia [21]. Projections indicate that one in five U.S. residents will be aged 65 years or older in 2030 [15], and that 34% of adults aged 65 years or older will belong to racially and ethnically minoritized groups in 2040 [22]. As the U.S. population ages and its demographic makeup shifts, it is increasingly important to examine the potential drivers of disparities in cognitive function and, in particular, to elucidate the contributions of everyday experiences of discrimination to cognitive health in later life.

Although existing research suggests that discrimination may contribute to cognitive disparities, less is known about these relationships longitudinally across diverse racial and ethnic groups in nationally representative samples. To address this gap, the present study uses longitudinal data from a large, representative cohort and includes analyses stratified by race and ethnicity to examine associations between perceived discrimination and cognitive outcomes.

## Methods

### Study Population and data Sources

This study used data from the Health and Retirement Study (HRS), a nationally representative longitudinal study of health and aging among U.S. adults aged 50 years and older and their spouses. The HRS began in 1992 and has been conducted biennially since 1994. The HRS is sponsored by the National Institute on Aging (grant number NIA U01AG009740) and is conducted by the University of Michigan.

Beginning in 2006, HRS implemented an enhanced face-to-face interview design in which a rotating half-sample of participants completed additional assessments, including a psychosocial Leave-Behind questionnaire. The remaining half-sample completed these assessments in 2008, and this rotating design continued in later waves. Perceived discrimination was measured through the Leave-Behind questionnaire; consequently, respondents were generally eligible to contribute discrimination data at approximately four-year intervals. Personwave records outside assigned Leave-Behind waves were considered structurally unavailable by design rather than missing due to item nonresponse and were not treated as missing observations [23].

We used pooled longitudinal data from 2008 to 2022. The 2006 wave was excluded to avoid combining the five-item discrimination scale used in 2006 with the six-item scale available from 2008 onward, which included the healthcare discrimination item. The analytic frame was restricted to respondents who were eligible for the Leave-Behind questionnaire and returned questionnaire data in at least one included wave. The 2022 Early Generation X entry cohort was excluded because the corresponding sampling weights were not yet available.

### Measures

#### Outcome Variables

The primary outcomes were global cognition and episodic memory scores, assessed using the adapted Telephone Interview for Cognitive Status (TICS) [24]. The global cognition score reflects overall cognitive performance across multiple cognitive domains, whereas the episodic memory score is based on immediate and delayed word recall tasks.

##### Exposure Variable

The primary exposure was perceived everyday discrimination, measured using the six-item Everyday Discrimination Scale [18] administered as part of the HRS Leave-Behind questionnaire from 2008 onward. The scale assesses the frequency of routine experiences of unfair treatment, including being treated with less courtesy or respect, receiving poorer service in restaurants or stores, being treated as less intelligent, being treated as feared, being threatened or harassed, and receiving poorer service or treatment from doctors or hospitals. Response options ranged from 1 (“almost every day”) to 6 (“never”). Items were reverse-coded and rescaled to range from 0 to 5, with higher values indicating more frequent perceived discrimination. Consistent with HRS scoring guidance, all six items were reverse-coded and averaged to create the perceived everyday discrimination score. The score was set to missing when more than three of the six items were missing; equivalently, the score was calculated when at least three items were non-missing [23].

#### Covariates

Covariates were selected a priori based on prior literature on perceived discrimination, cognitive aging, and later-life health [5, 6, 16, 25, 26]. Sociodemographic covariates included age at interview, sex, race and ethnicity, and educational attainment. Race and ethnicity were categorized as Non-Hispanic White, Non-Hispanic Black, Non-Hispanic Other, and Hispanic. In the public-use HRS race variable in this analysis, the masked Other race category includes American Indian, Alaska Native, Asian, Native Hawaiian, Pacific Islander, and other specified race responses, with categories collapsed to protect participant confidentiality [27]. The specific racial identities represented within this category could not be distinguished using the public-use race variable included in this analysis. Educational attainment was categorized as less than high school, high school, some college, and college graduate. Health covariates included body mass index (BMI) and self-reported physician diagnoses of stroke, heart disease, memory-related disease, hypertension, and diabetes. Survey wave was included in all models to account for secular trends and wave-specific differences in data collection.

### Statistical Analyses

Weighted descriptive statistics were used to summarize participant characteristics. Baseline characteristics were defined at each respondent’s first included observation. Pooled longitudinal characteristics are presented separately in the supplement.

We estimated population-averaged associations between perceived everyday discrimination and cognitive outcomes using weighted generalized estimating equations with an identity link and Gaussian family. Weighted generalized estimating equations were selected because the estimand of interest was the marginal association between perceived discrimination and cognitive function in the HRS target population, rather than subject-specific cognitive trajectories. Models specified an exchangeable working correlation structure for repeated observations within respondents and included survey wave as a categorical covariate. Survey weights, strata, and primary sampling units were incorporated through the survey design specification, and robust sandwich standard errors were used for inference.

To address potential bias from attrition and Leave-Behind nonresponse, longitudinal inverse probability weights were constructed beginning with the HRS survey weight from the first wave in which a respondent became eligible for the analytic sample. For subsequent waves, weights were adjusted using inverse probability weighting in two stages. First, among eligible respondent-wave records, we modeled the probability of completing the core interview. Second, among respondents who were eligible for the Leave-Behind questionnaire and completed the core interview, we modeled the conditional probability of returning Leave-Behind questionnaire data with a valid perceived discrimination score. The logistic models included demographic characteristics, prior-wave health indicators, prior-wave cognitive score, prior-wave perceived discrimination score, and survey wave. Final combined inverse probability weights were truncated at the 5th and 95th percentiles to reduce the influence of extreme weights.

After applying longitudinal inverse probability weights for attrition and Leave-Behind nonresponse, observations missing outcome, exposure, or covariate information required for a given model were excluded. The number of design-eligible records, core interview completions, Leave-Behind completions, exclusions due to missing variables, final analytic observations, and unique participants are reported in the Results and supplemental tables. All analyses were conducted in R.

## Results

Across the 2008 through 2022 waves, there were 99,140 design-eligible respondent-wave records based on HRS Leave-Behind eligibility and cohort entry. Of these, 67,218 completed self-core interviews, and 48,483 completed the Leave-Behind questionnaire. After excluding 1,379 records without required survey weight or design information, the eligible analytic frame included 47,104 respondent-wave records with valid survey weight and design information. We then excluded 1,500 records with missing outcome or exposure data and 515 records with missing covariate data, yielding a final analytic sample of 45,089 observations from 20,020 unique participants. Among the 47,104 respondent-wave records in the eligible analytic frame, only 2,015 (4.3%) were excluded because of missing outcome, exposure, or covariate information. Supplementary Table 1 summarizes wave-specific eligibility, self-core interview completion, Leave-Behind completion, variable-level missingness, missing survey weight/design information, and final analytic observations.

Table 1 presents weighted baseline characteristics measured at each participant’s first eligible analytic observation. The mean age at entry was 64.12 years. The mean global cognition score was 15.71, the mean episodic memory score was 10.15, and the mean perceived discrimination score was 0.62. The sample was 51.60% female and 48.40% male. Most participants were Non-Hispanic White, followed by Non-Hispanic Black, Hispanic, and Non-Hispanic Other participants. At baseline, 7.06% had a history of stroke, 21.05% had heart disease, 2.37% had memory-related disease, 54.38% had hypertension, and 21.47% had diabetes. Weighted characteristics of the pooled respondent-wave sample are shown in Supplementary Table 2.

Table 2 summarizes perceived discrimination by demographic and cohort characteristics. Mean perceived discrimination scores varied across sex, racial and ethnic groups, and birth cohorts. Non-Hispanic Black participants had the highest mean perceived discrimination score, followed by Non-Hispanic Other participants. Non-Hispanic White and Hispanic participants reported lower mean scores. Later birth cohorts reported higher mean perceived discrimination than earlier cohorts.

Table 3 presents adjusted associations between perceived discrimination and global cognition. In the full sample, higher perceived discrimination was associated with lower global cognition (estimate = −0.43; 95% CI: −0.52, −0.33). The association was also observed in sex-stratified models, with estimates of −0.34 among female participants and −0.51 among male participants. Although covariate estimates are shown for transparency, interpretation focuses on perceived discrimination, the primary exposure of interest.

Table 4 presents adjusted associations between perceived discrimination and episodic memory. In the full sample, higher perceived discrimination was associated with lower episodic memory (estimate = −0.30; 95% CI: −0.37, −0.24). Similar inverse associations were observed among female participants and male participants. Full covariate estimates are shown for transparency and are not interpreted as primary findings.

Tables 5 and 6 present race- and ethnicity-stratified associations. For global cognition, point estimates were inverse across all racial and ethnic groups, although confidence intervals included the null for Non-Hispanic Other participants. Estimates ranged from −0.33 among Non-Hispanic Other participants to −0.47 among Hispanic participants. For episodic memory, point estimates were also inverse across racial and ethnic groups, with confidence intervals including the null for Non-Hispanic Other participants. Estimates were broadly similar in direction across groups, although precision varied. Supplementary Tables 3 and 4 present cohort-stratified models. Across cohorts, perceived discrimination was generally inversely associated with global cognition and episodic memory, although the magnitude and precision of estimates varied across cohorts.

## Discussion

Perceived everyday discrimination was inversely associated with both global cognition and episodic memory in the full sample and in sex-stratified analyses. In race- and ethnicity-stratified analyses, point estimates were inverse across all groups, although confidence intervals for NH Other participants included the null for both outcomes. NH Black and NH Other participants reported higher average discrimination exposure than NH White and Hispanic participants. These findings indicate that perceived everyday discrimination is socially patterned and associated with cognitive performance in the overall older adult population, while the stratum-specific estimates for NH Other participants were less precise.

## Perceived Everyday Discrimination and Cognitive Outcomes

The inverse associations between perceived everyday discrimination and global cognition, and episodic memory observed in the full sample extend prior longitudinal evidence linking chronic psychosocial stressors to poorer cognitive performance in later life [6, 21, 25]. A one-unit higher perceived everyday discrimination score was associated with a 0.43-point lower global cognition score and a 0.30-point lower episodic memory score. The standard deviation of global cognition in this sample was 4.29 points, and that of episodic memory was 3.34 points, placing the discrimination-associated differences at approximately 10% of a standard deviation for each outcome, a conventional metric for contextualizing effect magnitude in cognitive aging research [28, 29]. For context, the adjusted global cognition coefficient for a self-reported diagnosis of memory disease in the same models was −3.93 points. Given the higher mean perceived discrimination scores observed among NH Black and NH Other adults, even modest associations at the individual level may have population-health relevance [30].

The episodic memory findings are noteworthy. Episodic memory is among the cognitive domains most sensitive to early aging-related change and serves as a reliable early marker of elevated dementia risk [31, 32]. Performance on delayed word recall tasks, specifically, has been shown to detect pathological cognitive trajectories years before clinical diagnosis [33]. The observed association between perceived everyday discrimination and lower episodic memory warrants attention given the relevance of this cognitive domain to early aging-related change, although the present study does not establish perceived discrimination as a marker of future cognitive decline.

Although this study was not designed to evaluate biological or neurobiological mechanisms, prior research has proposed that chronic exposure to psychosocial stressors may affect cognitive function through sustained activation of the hypothalamic-pituitary-adrenal axis, dysregulation of inflammatory signaling, and acceleration of biological aging processes [16, 20]. Neuroimaging and epigenetic studies have reported associations between discrimination exposure and reduced cortical integrity, altered neural connectivity, and markers of accelerated cellular aging [34–36]. The present findings are consistent with these proposed pathways, though the analytic approach was not structured to test them, and such consistency does not constitute evidence of mechanism.

## Racial and Ethnic Patterns in Exposure and Association

NH Black participants reported the highest mean perceived everyday discrimination scores (0.75), followed closely by NH Other participants (0.74), whereas NH White participants and Hispanic participants reported markedly lower scores (0.53 and 0.58, respectively). These exposure differences align with an extensive body of literature documenting racial and ethnic disparities in the frequency of perceived unfair treatment in the U.S [16, 20]. The adjusted difference in perceived everyday discrimination between Hispanic and NH White participants was small, a finding that should be interpreted within the broader social and demographic context of cognitive aging [5].

In race- and ethnicity-stratified models, inverse associations between perceived everyday discrimination and both cognitive outcomes were observed among NH White, NH Black, and Hispanic participants. Confidence intervals for the NH Other stratum included the null for both outcomes. This stratum was smaller than the other racial and ethnic groups, and its estimates were less precise. The category also aggregates several distinct racial populations, limiting interpretation of a single category-specific estimate [27]. The analyses did not formally test differences between stratum-specific coefficients.

In adjusted models, NH Black, NH Other, and Hispanic participants demonstrated lower mean cognitive scores relative to NH White participants across both outcomes, consistent with well-documented racial and ethnic disparities in later-life cognitive function [5, 6]. The present analyses were not designed to decompose the sources of these group differences. Prior research has attributed racial and ethnic disparities in cognitive aging to a broad set of interrelated factors, including differential educational quality and access, cumulative health disadvantage, and differential exposure to adverse social conditions across the life course [35–39].

## Cohort Variation in Perceived Discrimination

Adjusted perceived discrimination scores increased monotonically from earlier to later birth cohorts, from the AHEAD cohort (born before 1924) through the Late Baby Boomer cohort (born 1960 to 1965). This gradient may reflect cohort-specific differences in willingness to report unfair treatment, the salience with which such experiences are recognized, or genuine differences in the frequency of exposure across historical periods [20]. Cohort-stratified associations between perceived discrimination and cognitive outcomes are presented descriptively in Supplementary Tables 3 and 4. Variation in sample size and precision across cohorts precludes substantive conclusions about cohort differences in the discrimination-cognition relationship, and these findings are reported for transparency rather than as primary results.

## Sex Differences

Inverse associations between perceived everyday discrimination and cognitive performance were observed for both women and men. Point estimates were somewhat larger among men than women for global cognition (male: −0.51; female: −0.34) and episodic memory (male: −0.39; female: −0.21), though confidence intervals overlapped and the study was neither designed nor powered to formally test for effect modification by sex. Women reported lower mean perceived everyday discrimination than men in this cohort (0.51 vs. 0.62) yet demonstrated modestly higher mean cognitive scores in adjusted models, a pattern consistent with prior research on sex differences in cognitive performance in later life [40]. These patterns are characterized descriptively; the present analyses do not assess the mechanisms underlying sex differences in cognitive performance or in discrimination exposure.

## Implications

The findings support considering perceived everyday discrimination as one of several psychosocial stressors relevant to cognitive health in later life. Future cognitive aging research can extend this evidence by measuring experiences of unfair treatment directly rather than inferring them from racial or ethnic group membership and by examining the frequency, attribution, timing, and cumulative burden of these experiences [41, 42].

During psychosocial assessment, clinicians may ask whether experiences of unfair treatment contribute to distress, affect healthcare interactions, or create barriers to care. When such concerns are identified, responses may include supportive discussion and, with the patient’s agreement, connection with relevant social or community resources. Health systems may support these responses by training staff to conduct sensitive, non-stigmatizing assessments and by establishing referral relationships and follow-up procedures [43]. The present study did not evaluate routine screening for perceived discrimination, enhanced cognitive monitoring based on discrimination scores, or whether cognitive assessment instruments perform equivalently across racial and ethnic groups; these issues require separate evaluation.

## Strengths, Limitations, and Future Directions

This study has several notable strengths. The HRS provides a large, diverse, and nationally representative sample with repeated cognitive assessments; the present analyses drew on eight biennial waves spanning 14 years, from 2008 to 2022, supporting longitudinal analyses with race- and sex-stratified estimates. Weighted generalized estimating equation models incorporated the complex sampling design and longitudinal inverse probability weights to address attrition and Leave-Behind nonresponse. After adjustment for attrition and Leave-Behind nonresponse using longitudinal inverse probability weighting, 4.3% of eligible analytic records were excluded due to missing outcome, exposure, or covariate information. Consistent use of the six-item Everyday Discrimination Scale from 2008 onward, with adherence to HRS scoring guidance for missing items, supported measurement comparability across waves. Convergent inverse associations across two distinct cognitive outcomes and across multiple demographic strata strengthen confidence in the reliability of the findings.

Several limitations are important to acknowledge. Perceived discrimination was measured via self-report and may be subject to reporting bias or differential response patterns across groups [42, 44]. The Everyday Discrimination Scale captures the frequency of perceived routine unfair treatment without attribution items in the present analyses; accordingly, these findings are not interpreted as estimates of race-attributed or any other specific form of discrimination. Cognitive function was assessed using brief performance-based instruments rather than clinically adjudicated diagnoses, and some misclassification is possible. The study estimand is associative, and the design does not support causal inference. Residual confounding by unmeasured social, psychological, or health-related factors may persist despite extensive covariate adjustment. The discrimination measure reflects recent experiences and may not adequately capture the cumulative or life-course burden of perceived unfair treatment [41, 42].

Future research would benefit from formally specifying causal estimands and the identifying assumptions required for causal inference, rather than relying on adjustment alone. Incorporating biological markers of stress physiology and neuroimaging indicators of brain health would allow more direct evaluation of proposed mechanistic pathways. Studies using the full attribution items available in the HRS Leave-Behind questionnaire could examine whether associations differ by the perceived basis of unfair treatment. Linking observed differences in continuous cognitive performance to clinically adjudicated dementia outcomes would help determine whether the associations documented here translated into differential risk for cognitive impairment and dementia in later life.

## Conclusions

In this nationally representative longitudinal study of older U.S. adults, each one-unit increase in perceived everyday discrimination was associated with a 0.43-point decrease in global cognition and a 0.30-point decrease in episodic memory, representing differences of approximately 10% of a standard deviation for each outcome. NH Black and NH Other participants reported markedly higher average perceived discrimination than NH White participants, yet associations with cognitive outcomes were broadly consistent in direction across racial and ethnic groups. These findings identify perceived everyday discrimination as a socially patterned correlate of cognitive health in aging populations and add longitudinal population-based evidence to the literature on associations between chronic exposure to social stressors and cognitive health in later life.

## Supplementary Material

Supplementary Tables

The online version contains supplementary material available at https://doi.org/10.1007/s40615-026-03174-w.

## Figures and Tables

**Table 1:** Weighted baseline characteristics at participants’ first eligible analytic observation, Health and Retirement Study, 2008–2022

Characteristic	*n*, weighted mean (SD), or weighted percentage (95% CI)
Number of unique participants	20,020
Global cognitive score	15.71 (4.29)
Episodic memory score	10.15 (3.34)
Perceived discrimination	0.62 (0.76)
Age at baseline	64.12 (9.69)
Sex	
Male	48.40 (47.38, 49.43)
Female	51.60 (50.57, 52.62)
Race and ethnicity	
Non-Hispanic White	73.35 (70.72, 75.98)
Non-Hispanic Black	10.98 (9.85, 12.12)
Non-Hispanic Other	5.33 (4.37, 6.29)
Hispanic	10.34 (7.71, 12.97)
Education	
Less than high school	14.18 (12.44, 15.92)
High school	31.51 (30.12, 32.90)
Some college	26.53 (25.05, 28.01)
College graduate	27.77 (25.89, 29.66)
Cohort	
HRS (born 1931–1941)	21.19 (19.81, 22.56)
AHEAD (born < 1924)	4.45 (3.94, 4.96)
CODA (born 1924–1930)	4.01 (3.43, 4.60)
War Baby (born 1942–1947)	15.02 (13.93, 16.10)
Early Baby Boomer (born 1948–1953)	19.30 (17.63, 20.98)
Mid Baby Boomer (born 1954–1959)	20.26 (17.67, 22.84)
Late Baby Boomer (born 1960–1965)	15.77 (13.62, 17.92)
BMI	28.85 (6.06)
Health Conditions	
Stroke	7.06 (6.30, 7.82)
Heart disease	21.05 (19.89, 22.20)
Memory disease	2.37 (1.81, 2.92)
High blood pressure	54.38 (52.83, 55.93)
Diabetes	21.47 (20.08, 22.85)

Continuous variables are presented as weighted means with standard deviations; categorical variables are presented as weighted percentages with 95% confidence intervals. Estimates reflect each participant’s first eligible analytic observation and incorporate the HRS sampling design and survey weight

**Table 2:** Descriptive means and adjusted differences in perceived discrimination

	Unadjusted mean (SE)	Adjusted difference (95% CI)
Sex		
Male	0.62 (0.01)	Reference
Female	0.51 (0.01)	−0.09 (−0.12, −0.06)
Race and ethnicity		
Non-Hispanic White	0.53 (0.01)	Reference
Non-Hispanic Black	0.75 (0.02)	0.19 (0.13, 0.24)
Non-Hispanic Other	0.74 (0.04)	0.14 (0.05, 0.23)
Hispanic	0.58 (0.03)	−0.01 (−0.08, 0.05)
Education		
Less than high school	0.61 (0.03)	Reference
High school	0.55 (0.01)	−0.06 (−0.12, −0.01)
Some college	0.60 (0.01)	−0.05 (−0.11, 0.01)
College graduate	0.53 (0.01)	−0.13 (−0.18, −0.07)
Cohort		
HRS (born 1931–1941)	0.45 (0.01)	Reference
AHEAD (born < 1924)	0.37 (0.02)	−0.08 (−0.13, −0.03)
CODA (born 1924–1930)	0.42 (0.03)	−0.03 (−0.10, 0.05)
War Baby (born 1942–1947)	0.49 (0.02)	0.05 (0.01, 0.08)
Early Baby Boomer (born 1948–1953)	0.60 (0.01)	0.15 (0.11, 0.18)
Mid Baby Boomer (born 1954–1959)	0.71 (0.03)	0.27 (0.21, 0.33)
Late Baby Boomer (born 1960–1965)	0.76 (0.04)	0.34 (0.27, 0.42)

The unadjusted mean column displays weighted mean perceived discrimination scores with standard errors. The adjusted difference column displays adjusted mean differences in perceived discrimination relative to the reference group. Models adjust for sex, race and ethnicity, education, cohort, and survey wave. Analyses incorporate the HRS sampling design, survey weights, longitudinal inverse probability weights, and repeated observations within respondents

**Table 3:** Adjusted associations of perceived discrimination with global cognition, stratified by sex

	Full Sample Estimate (95% CI)	Male Estimate (95% CI)	Female Estimate (95% CI)
Perceived discrimination	−0.43 (−0.52, −0.33)	−0.51 (−0.64, −0.38)	−0.34 (−0.47, −0.21)
Age	−0.13 (−0.13, −0.12)	−0.13 (−0.14, −0.12)	−0.13 (−0.14, −0.11)
Sex			
Male	Reference		
Female	0.58 (0.44, 0.72)		
Race and ethnicity			
Non-Hispanic White	Reference	Reference	Reference
Non-Hispanic Black	−2.36 (−2.61, −2.11)	−2.04 (−2.35, −1.72)	−2.57 (−2.93, −2.22)
Non-Hispanic Other	−1.44 (−1.85, −1.03)	−1.13 (−1.67, −0.58)	−1.74 (−2.32, −1.16)
Hispanic	−1.24 (−1.52, −0.96)	−0.95 (−1.36, −0.53)	−1.52 (−1.87, −1.17)
Education			
Less than high school	Reference	Reference	Reference
High school	2.27 (2.02, 2.51)	2.11 (1.73, 2.48)	2.38 (2.08, 2.67)
Some college	3.31 (3.07, 3.56)	3.13 (2.73, 3.53)	3.44 (3.15, 3.74)
College graduate	4.62 (4.38, 4.87)	4.51 (4.13, 4.89)	4.70 (4.42, 4.98)
BMI	0.03 (0.02, 0.04)	0.05 (0.03, 0.07)	0.03 (0.02, 0.04)
Stroke	−0.81 (−1.09, −0.53)	−0.76 (−1.20, −0.33)	−0.87 (−1.21, −0.53)
Heart disease	−0.18 (−0.36, 0.00)	−0.27 (−0.57, 0.03)	−0.05 (−0.25, 0.14)
Memory disease	−3.93 (−4.50, −3.36)	−4.18 (−4.93, −3.44)	−3.59 (−4.25, −2.92)
High blood pressure	−0.29 (−0.46, −0.12)	−0.16 (−0.39, 0.07)	−0.41 (−0.66, −0.17)
Diabetes	−0.32 (−0.51, −0.13)	−0.22 (−0.56, 0.11)	−0.41 (−0.61, −0.22)

Estimates (95% CI) are fully adjusted marginal population-averaged coefficients from weighted generalized estimating equation models. Models adjust for all listed variables and survey wave, which is not shown. Analyses incorporate the HRS sampling design, survey weights, longitudinal inverse probability weights for attrition and Leave-Behind nonresponse, and repeated observations within respondents

**Table 4:** Adjusted associations of perceived discrimination with episodic memory, stratified by Sex

	Total Sample Estimate (95% CI)	Male Estimate (95% CI)	Female Estimate (95% CI)
Perceived discrimination	−0.30 (−0.37, −0.24)	−0.39 (−0.49, −0.30)	−0.21 (−0.32, −0.11)
Age	−0.11 (−0.11, −0.10)	−0.11 (−0.12, −0.10)	−0.11 (−0.11, −0.10)
Sex			
Male	Reference		
Female	0.97 (0.86, 1.08)		
Race and ethnicity			
Non-Hispanic White	Reference	Reference	Reference
Non-Hispanic Black	−1.30 (−1.49, −1.10)	−1.02 (−1.27, −0.77)	−1.48 (−1.74, −1.21)
Non-Hispanic Other	−0.97 (−1.26, −0.67)	−0.75 (−1.18, −0.32)	−1.18 (−1.58, −0.79)
Hispanic	−0.68 (−0.87, −0.48)	−0.56 (−0.85, −0.27)	−0.78 (−1.03, −0.54)
Education			
Less than high school	Reference	Reference	Reference
High school	1.07 (0.88, 1.25)	1.02 (0.75, 1.29)	1.11 (0.88, 1.34)
Some college	1.77 (1.58, 1.96)	1.73 (1.45, 2.01)	1.81 (1.56, 2.06)
College graduate	2.67 (2.48, 2.87)	2.67 (2.39, 2.94)	2.68 (2.44, 2.92)
BMI	0.02 (0.01, 0.03)	0.03 (0.01, 0.05)	0.02 (0.01, 0.03)
Stroke	−0.62 (−0.85, −0.39)	−0.73 (−1.10, −0.37)	−0.52 (−0.77, −0.27)
Heart disease	−0.16 (−0.31, −0.02)	−0.20 (−0.42, 0.01)	−0.10 (−0.28, 0.08)
Memory disease	−2.80 (−3.21, −2.39)	−3.05 (−3.56, −2.54)	−2.48 (−2.97, −1.98)
High blood pressure	−0.18 (−0.31, −0.04)	−0.10 (−0.29, 0.09)	−0.26 (−0.47, −0.06)
Diabetes	−0.21 (−0.35, −0.06)	−0.10 (−0.35, 0.15)	−0.32 (−0.47, −0.16)

Estimates (95% CI) are fully adjusted marginal population-averaged coefficients from weighted generalized estimating equation models. Models adjust for all listed variables and survey wave, which is not shown. Analyses incorporate the HRS sampling design, survey weights, longitudinal inverse probability weights for attrition and Leave-Behind nonresponse, and repeated observations within respondents

**Table 5:** Adjusted associations of perceived discrimination with global cognition, stratified by race/ethnicity

	Non-Hispanic White Estimate (95% CI)	Non-Hispanic Black Estimate (95% CI)	Non-Hispanic Other Estimate (95% CI)	Hispanic Estimate (95% CI)
Perceived discrimination	−0.42 (−0.53, −0.31)	−0.43 (−0.62, −0.23)	−0.33 (−0.76, 0.11)	−0.47 (−0.66, −0.28)
Age	−0.13 (−0.14, −0.12)	−0.12 (−0.14, −0.10)	−0.11 (−0.14, −0.07)	−0.10 (−0.13, −0.07)
Sex				
Male	Reference	Reference	Reference	Reference
Female	0.72 (0.53, 0.91)	0.03 (−0.33, 0.38)	0.18 (−0.46, 0.83)	−0.06 (−0.49, 0.37)
Education				
Less than high school	Reference	Reference	Reference	Reference
High school	2.33 (1.96, 2.70)	1.88 (1.29, 2.47)	2.90 (1.90, 3.90)	2.23 (1.65, 2.81)
Some college	3.29 (2.93, 3.65)	3.46 (2.80, 4.11)	4.67 (3.64, 5.71)	2.95 (2.24, 3.66)
College graduate	4.59 (4.22, 4.95)	5.00 (4.51, 5.50)	5.96 (4.83, 7.08)	4.26 (3.59, 4.93)
BMI	0.03 (0.02, 0.05)	0.06 (0.04, 0.08)	−0.02 (−0.08, 0.04)	0.04 (0.01, 0.08)
Stroke	−0.89 (−1.21, −0.58)	−0.53 (−1.17, 0.12)	−0.28 (−1.21, 0.66)	−0.70 (−1.58, 0.18)
Heart disease	−0.10 (−0.32, 0.11)	−0.44 (−0.89, 0.02)	−0.50 (−1.25, 0.25)	−0.11 (−0.76, 0.54)
Memory disease	−4.59 (−5.13, −4.05)	−2.80 (−4.57, −1.03)	−1.98 (−3.36, −0.60)	−2.77 (−3.91, −1.63)
High blood pressure	−0.27 (−0.46, −0.08)	−0.11 (−0.52, 0.29)	−0.18 (−1.03, 0.68)	−0.45 (−0.91, 0.01)
Diabetes	−0.41 (−0.65, −0.16)	−0.14 (−0.57, 0.29)	−0.30 (−0.99, 0.40)	−0.17 (−0.61, 0.28)

Estimates (95% CI) are fully adjusted marginal population-averaged coefficients from weighted generalized estimating equation models. Models adjust for all listed variables and survey wave, which is not shown. Analyses incorporate the HRS sampling design, survey weights, longitudinal inverse probability weights for attrition and Leave-Behind nonresponse, and repeated observations within respondents

**Table 6:** Adjusted associations of perceived discrimination with episodic memory, stratified by race/ethnicity

Variable	Non-Hispanic White Estimate (95% CI)	Non-Hispanic Black Estimate (95% CI)	Non-Hispanic Other Estimate (95% CI)	Hispanic Estimate (95% CI)
Perceived discrimination	−0.33 (−0.41, −0.25)	−0.28 (−0.44, −0.12)	−0.24 (−0.60, 0.12)	−0.21 (−0.36, −0.07)
Age	−0.11 (−0.12, −0.10)	−0.10 (−0.11, −0.08)	−0.10 (−0.12, −0.07)	−0.09 (−0.11, −0.07)
Sex				
Male	Reference	Reference	Reference	Reference
Female	1.06 (0.92, 1.20)	0.53 (0.25, 0.82)	0.64 (0.15, 1.13)	0.73 (0.40, 1.06)
Education				
Less than high school	Reference	Reference	Reference	Reference
High school	1.05 (0.78, 1.33)	0.92 (0.51, 1.32)	1.42 (0.75, 2.08)	1.27 (0.85, 1.68)
Some college	1.74 (1.46, 2.02)	1.86 (1.42, 2.30)	2.38 (1.63, 3.14)	1.71 (1.20, 2.22)
College graduate	2.65 (2.37, 2.93)	2.65 (2.31, 3.00)	3.35 (2.58, 4.12)	2.53 (1.97, 3.10)
BMI	0.02 (0.01, 0.03)	0.04 (0.02, 0.05)	−0.00 (−0.05, 0.05)	0.03 (0.01, 0.05)
Stroke	−0.66 (−0.94, −0.38)	−0.45 (−0.84, −0.06)	−0.48 (−1.12, 0.17)	−0.53 (−1.21, 0.15)
Heart disease	−0.11 (−0.28, 0.06)	−0.31 (−0.63, 0.01)	−0.50 (−1.04, 0.05)	−0.17 (−0.64, 0.29)
Memory disease	−3.34 (−3.70, −2.99)	−1.90 (−2.92, −0.89)	−0.87 (−1.89, 0.16)	−1.95 (−2.69, −1.21)
High blood pressure	−0.18 (−0.34, −0.02)	0.01 (−0.32, 0.34)	0.11 (−0.64, 0.86)	−0.27 (−0.58, 0.03)
Diabetes	−0.23 (−0.42, −0.03)	−0.24 (−0.54, 0.07)	−0.34 (−0.87, 0.19)	−0.11 (−0.39, 0.16)

Estimates (95% CI) are fully adjusted marginal population-averaged coefficients from weighted generalized estimating equation models. Models adjust for all listed variables and survey wave, which is not shown. Analyses incorporate the HRS sampling design, survey weights, longitudinal inverse probability weights for attrition and Leave-Behind nonresponse, and repeated observations within respondents

## Data Availability

This study used data from the Health and Retirement Study (HRS), a publicly available dataset produced and distributed by the University of Michigan with funding from the National Institute on Aging. All data used in this analysis, including the 2022 h Core (Final) public use dataset and the HRS Psychosocial and Lifestyle Questionnaire 2006–2022, are publicly available to registered researchers through the HRS data portal at https://hrs.isr.umich.edu. The HRS (Health and Retirement Study) is sponsored by the National Institute on Aging (grant number NIA U01AG009740) and is conducted by the University of Michigan.
