# Supplementary material for: Perceived Discrimination and Cognitive Function Among Older Adults: An Analysis of the Health and Retirement Study
Source: J Racial Ethn Health Disparities. Author manuscript; Available in PMC 2026 Oct 5. (PMC13637015; doi:10.1007/s40615-026-03174-w)
Supplement: Supplementary file 1 [file NIHMS2211170-supplement-Supplementary_Tables.docx]

**Supplementary Table 1. Wave-specific analytic sample flow and variable-level missingness among Leave-Behind eligible respondent-wave records, Health and Retirement Study, 2008–2022**

| Year | Eligible Core | Completed Core | Completed LB | Miss Global Cog | Miss Epis Mem | Miss Discrim | Miss BMI | Miss Race | Miss Education | Miss Survey Wt/Design | Final Included |
| --- | --- | --- | --- | --- | --- | --- | --- | --- | --- | --- | --- |
| 2008 | 8296 | 7632 | 6645 | 178 | 178 | 88 | 75 | 2 | 1 | 0 | 6312 |
| 2010 | 11212 | 10366 | 7866 | 193 | 193 | 111 | 101 | 6 | 1 | 0 | 7461 |
| 2012 | 11842 | 9212 | 6956 | 138 | 138 | 143 | 85 | 10 | 1 | 217 | 6386 |
| 2014 | 11630 | 8726 | 7057 | 161 | 161 | 102 | 81 | 7 | 1 | 11 | 6712 |
| 2016 | 14197 | 9576 | 6132 | 0 | 0 | 103 | 63 | 11 | 1 | 432 | 5553 |
| 2018 | 13690 | 8294 | 5535 | 0 | 0 | 104 | 58 | 10 | 1 | 210 | 5165 |
| 2020 | 14352 | 6995 | 4467 | 123 | 123 | 67 | 46 | 7 | 0 | 337 | 3918 |
| 2022 | 13921 | 6417 | 3825 | 0 | 0 | 50 | 29 | 6 | 1 | 172 | 3582 |
| **Total** | **99140** | **67218** | **48483** | **793** | **793** | **768** | **538** | **59** | **7** | **1379** | **45089** |

Eligible Core Sample represents the maximum number of respondent-wave records eligible for inclusion based on HRS cohort membership, year of observation, and assignment to the rotating Leave-Behind questionnaire design. Respondents not assigned to the Leave-Behind questionnaire in a given wave were structurally unavailable by design and were not considered missing.

The Leave-Behind questionnaire is administered to a rotating half-sample of HRS respondents approximately every four years. Consequently, differences between the Eligible Core Sample and Completed Leave-Behind Questionnaire counts primarily reflect study design and survey participation rather than item-level missingness.

Missingness counts represent records with unavailable values among respondents completing the Leave-Behind questionnaire. Age, sex, and medical history variables had no missing data after HRS/RAND processing and are therefore not shown.

The final analytic sample excluded records with missing outcome, exposure, covariate, survey weight, or design information required for the weighted generalized estimating equation analyses.

The Early Generation X cohort introduced in 2022 was excluded because corresponding survey weights were not available at the time of analysis.

**Supplementary Table 2. Weighted characteristics of the pooled respondent-wave analytic sample, Health and Retirement Study, 2008–2022**

| Number of observations | 45,089 |
| --- | --- |
| Global cognitive score | 15.50 (4.32) |
| Episodic memory score | 9.94 (3.36) |
| Perceived discrimination | 0.56 (0.71) |
| Age at interview | 68.46 (9.99) |
| **Sex** |  |
| Male | 46.98 (46.11, 47.85) |
| Female | 53.02 (52.15, 53.89) |
| **Race and ethnicity** |  |
| Non-Hispanic White | 77.42 (74.92, 79.92) |
| Non-Hispanic Black | 9.69 (8.67, 10.72) |
| Non-Hispanic Other | 3.96 (3.35, 4.57) |
| Hispanic | 8.92 (6.60, 11.25) |
| **Education** |  |
| Less than high school | 12.78 (11.32, 14.24) |
| High school | 32.70 (31.33, 34.08) |
| Some college | 26.11 (24.86, 27.35) |
| College graduate | 28.41 (26.63, 30.18) |
| **Cohort** |  |
| HRS (born 1931-1941) | 23.90 (22.36, 25.45) |
| AHEAD (born < 1924) | 3.65 (3.14, 4.16) |
| CODA (born 1924-1930) | 4.17 (3.49, 4.85) |
| War Baby (born 1942-1947) | 18.58 (17.24, 19.93) |
| Early Baby Boomer (born 1948-1953) | 21.44 (19.73, 23.16) |
| Mid Baby Boomer (born 1954-1959) | 19.74 (17.00, 22.49) |
| Late Baby Boomer (born 1960-1965) | 8.50 (7.29, 9.71) |
| BMI | 28.59 (5.97) |
| **Health Conditions** |  |
| Stroke | 8.27 (7.71, 8.84) |
| Heart disease | 26.17 (25.28, 27.07) |
| Memory disease | 2.54 (2.18, 2.90) |
| High blood pressure | 60.10 (58.96, 61.24) |
| Diabetes | 25.25 (23.97, 26.53) |

Continuous variables are presented as weighted means with standard deviations; categorical variables are presented as weighted percentages with 95% confidence intervals. Estimates reflect the pooled longitudinal respondent-wave sample from the 2008–2022 HRS waves and incorporate the HRS sampling design and survey weights. Variance estimates account for repeated observations within respondents.

**Supplementary Table 3: Adjusted associations of perceived discrimination with global cognition, stratified by HRS birth cohort**

|  | HRS (born 1931-1941)  Estimate (95% CI) | AHEAD (born < 1924)  Estimate (95% CI) | CODA (born 1924-1930)  Estimate (95% CI) | War Baby (born 1942-1947)  Estimate (95% CI) | Early Baby Boomer (born 1948-1953)  Estimate (95% CI) | Mid Baby Boomer (born 1954-1959)  Estimate (95% CI) | Late Baby Boomer (born 1960-1965)  Estimate (95% CI) |
| --- | --- | --- | --- | --- | --- | --- | --- |
| Perceived discrimination | -0.23 (-0.36, -0.09) | -0.72 (-1.04, -0.39) | -0.34 (-0.64, -0.04) | -0.13 (-0.31, 0.05) | -0.31 (-0.48, -0.15) | -0.23 (-0.38, -0.09) | -0.46 (-0.72, -0.20) |
| Age | -0.15 (-0.17, -0.14) | -0.17 (-0.21, -0.13) | -0.16 (-0.21, -0.11) | -0.12 (-0.16, -0.08) | -0.10 (-0.14, -0.06) | -0.11 (-0.18, -0.04) | -0.00 (-0.10, 0.10) |
| **Sex** |  |  |  |  |  |  |  |
| Male | Reference | Reference | Reference | Reference | Reference | Reference | Reference |
| Female | 1.15 (1.00, 1.31) | 0.90 (0.46, 1.33) | 1.28 (0.93, 1.62) | 1.14 (0.83, 1.44) | 0.86 (0.61, 1.10) | 0.75 (0.43, 1.07) | 0.70 (0.30, 1.10) |
| **Race and ethnicity** |  |  |  |  |  |  |  |
| Non-Hispanic White | Reference | Reference | Reference | Reference | Reference | Reference | Reference |
| Non-Hispanic Black | -1.39 (-1.70, -1.07) | -1.02 (-1.63, -0.41) | -1.37 (-1.86, -0.89) | -0.89 (-1.35, -0.44) | -1.29 (-1.67, -0.91) | -1.48 (-1.93, -1.03) | -1.25 (-1.71, -0.79) |
| Non-Hispanic Other | -0.79 (-1.31, -0.26) | -0.61 (-1.50, 0.28) | -0.69 (-2.15, 0.77) | -1.31 (-1.91, -0.70) | -1.53 (-2.10, -0.96) | -1.02 (-1.50, -0.54) | -0.05 (-0.73, 0.63) |
| Hispanic | -0.71 (-0.98, -0.45) | -0.33 (-1.02, 0.36) | -0.59 (-1.86, 0.68) | -0.02 (-0.59, 0.56) | -0.87 (-1.32, -0.42) | -0.71 (-1.22, -0.20) | -0.54 (-1.15, 0.08) |
| **Education** |  |  |  |  |  |  |  |
| Less than high school | Reference | Reference | Reference | Reference | Reference | Reference | Reference |
| High school | 1.08 (0.86, 1.30) | 1.11 (0.50, 1.72) | 1.23 (0.58, 1.88) | 1.47 (0.99, 1.96) | 1.20 (0.76, 1.64) | 0.54 (-0.01, 1.09) | 1.05 (-0.01, 2.11) |
| Some college | 1.80 (1.56, 2.05) | 1.76 (1.17, 2.36) | 1.62 (0.86, 2.38) | 1.94 (1.36, 2.52) | 2.11 (1.59, 2.63) | 1.36 (0.84, 1.87) | 1.80 (0.65, 2.94) |
| College graduate | 2.53 (2.28, 2.78) | 2.09 (1.48, 2.69) | 2.05 (1.13, 2.97) | 3.23 (2.71, 3.74) | 2.90 (2.32, 3.47) | 2.33 (1.90, 2.77) | 2.65 (1.62, 3.69) |
| BMI | 0.01 (-0.01, 0.02) | 0.05 (0.01, 0.09) | 0.03 (-0.01, 0.06) | 0.02 (-0.01, 0.05) | 0.03 (0.01, 0.04) | 0.01 (-0.01, 0.03) | 0.03 (0.00, 0.06) |
| Stroke | -0.82 (-1.07, -0.56) | -0.33 (-0.87, 0.21) | 0.68 (-0.02, 1.39) | -0.22 (-0.86, 0.42) | -0.77 (-1.28, -0.25) | -0.77 (-1.24, -0.29) | -1.32 (-2.68, 0.03) |
| Heart disease | 0.03 (-0.18, 0.23) | 0.32 (-0.12, 0.75) | 0.15 (-0.33, 0.63) | -0.53 (-0.91, -0.14) | -0.32 (-0.65, 0.00) | -0.16 (-0.58, 0.25) | 0.26 (-0.41, 0.93) |
| Memory disease | -2.56 (-2.97, -2.15) | -2.89 (-3.57, -2.21) | -3.35 (-4.03, -2.68) | -2.89 (-4.76, -1.02) | -3.02 (-3.87, -2.17) | -2.62 (-3.86, -1.38) | -2.41 (-3.60, -1.23) |
| High blood pressure | -0.30 (-0.51, -0.09) | 0.03 (-0.39, 0.44) | 0.11 (-0.31, 0.53) | -0.26 (-0.57, 0.04) | -0.19 (-0.46, 0.09) | -0.19 (-0.56, 0.18) | 0.07 (-0.48, 0.63) |
| Diabetes | -0.05 (-0.33, 0.23) | -0.09 (-0.56, 0.37) | 0.30 (-0.17, 0.76) | -0.46 (-0.82, -0.11) | -0.24 (-0.55, 0.06) | -0.34 (-0.74, 0.07) | -0.33 (-0.74, 0.09) |

Note: Estimates (95% CI) are fully adjusted marginal population-averaged coefficients from weighted generalized estimating equation models. Models adjust for all listed variables and survey wave, not shown. Analyses account for the HRS sampling design, survey weights, longitudinal inverse probability weights, and repeated observations within respondents.

**Supplementary Table 4: Adjusted associations of perceived discrimination with episodic memory, stratified by HRS birth cohort**

|  | HRS (born 1931-1941)  Estimate (95% CI) | AHEAD (born < 1924)  Estimate (95% CI) | CODA (born 1924-1930)  Estimate (95% CI) | War Baby (born 1942-1947)  Estimate (95% CI) | Early Baby Boomer (born 1948-1953)  Estimate (95% CI) | Mid Baby Boomer (born 1954-1959)  Estimate (95% CI) | Late Baby Boomer (born 1960-1965)  Estimate (95% CI) |
| --- | --- | --- | --- | --- | --- | --- | --- |
| Perceived discrimination | -0.36 (-0.55, -0.17) | -0.92 (-1.30, -0.54) | -0.48 (-0.82, -0.14) | -0.28 (-0.51, -0.05) | -0.45 (-0.67, -0.24) | -0.28 (-0.47, -0.10) | -0.60 (-0.89, -0.31) |
| Age | -0.18 (-0.20, -0.16) | -0.20 (-0.26, -0.14) | -0.17 (-0.24, -0.11) | -0.16 (-0.22, -0.10) | -0.11 (-0.17, -0.06) | -0.14 (-0.23, -0.05) | -0.01 (-0.13, 0.12) |
| **Sex** |  |  |  |  |  |  |  |
| Male | Reference | Reference | Reference | Reference | Reference | Reference | Reference |
| Female | 0.74 (0.55, 0.93) | 0.14 (-0.50, 0.78) | 0.68 (0.23, 1.13) | 0.86 (0.46, 1.27) | 0.45 (0.12, 0.78) | 0.36 (0.01, 0.71) | 0.25 (-0.21, 0.72) |
| **Race and ethnicity** |  |  |  |  |  |  |  |
| Non-Hispanic White | Reference | Reference | Reference | Reference | Reference | Reference | Reference |
| Non-Hispanic Black | -2.45 (-2.89, -2.02) | -2.69 (-3.49, -1.90) | -2.80 (-3.58, -2.02) | -1.93 (-2.62, -1.23) | -2.22 (-2.76, -1.69) | -2.65 (-3.21, -2.10) | -2.17 (-2.76, -1.58) |
| Non-Hispanic Other | -0.98 (-1.83, -0.13) | -2.02 (-4.35, 0.30) | -1.01 (-3.53, 1.51) | -1.78 (-2.79, -0.78) | -2.35 (-3.19, -1.52) | -1.64 (-2.20, -1.09) | -0.26 (-1.08, 0.56) |
| Hispanic | -1.18 (-1.62, -0.73) | -1.26 (-2.17, -0.35) | -0.93 (-2.83, 0.96) | -0.56 (-1.43, 0.31) | -1.41 (-1.90, -0.91) | -1.47 (-2.22, -0.72) | -1.12 (-1.83, -0.40) |
| **Education** |  |  |  |  |  |  |  |
| Less than high school | Reference | Reference | Reference | Reference | Reference | Reference | Reference |
| High school | 2.30 (1.92, 2.69) | 2.17 (1.31, 3.04) | 2.86 (2.17, 3.54) | 2.80 (2.11, 3.48) | 2.31 (1.63, 2.99) | 1.63 (0.92, 2.34) | 2.11 (0.57, 3.65) |
| Some college | 3.33 (3.03, 3.63) | 3.12 (2.30, 3.94) | 3.47 (2.62, 4.32) | 3.48 (2.68, 4.28) | 3.60 (2.86, 4.33) | 2.90 (2.18, 3.62) | 3.28 (1.64, 4.93) |
| College graduate | 4.41 (4.05, 4.76) | 3.59 (2.75, 4.44) | 4.31 (3.37, 5.26) | 5.38 (4.64, 6.11) | 4.87 (4.06, 5.69) | 4.15 (3.53, 4.76) | 4.48 (3.01, 5.95) |
| BMI | 0.02 (-0.01, 0.04) | 0.06 (0.01, 0.11) | 0.04 (-0.01, 0.10) | 0.05 (0.01, 0.09) | 0.04 (0.02, 0.06) | 0.02 (-0.00, 0.05) | 0.05 (0.01, 0.08) |
| Stroke | -1.13 (-1.46, -0.80) | -0.60 (-1.33, 0.13) | 0.66 (-0.03, 1.35) | -0.34 (-1.12, 0.44) | -0.94 (-1.58, -0.30) | -0.89 (-1.58, -0.21) | -1.39 (-3.04, 0.26) |
| Heart disease | 0.10 (-0.14, 0.34) | 0.21 (-0.39, 0.80) | 0.26 (-0.28, 0.79) | -0.44 (-0.93, 0.04) | -0.42 (-0.83, -0.02) | -0.24 (-0.67, 0.20) | 0.14 (-0.67, 0.95) |
| Memory disease | -3.53 (-4.14, -2.91) | -4.51 (-5.59, -3.43) | -5.43 (-6.64, -4.22) | -4.08 (-6.53, -1.64) | -4.72 (-5.81, -3.63) | -3.16 (-4.91, -1.40) | -2.81 (-4.19, -1.43) |
| High blood pressure | -0.44 (-0.74, -0.15) | 0.18 (-0.35, 0.72) | -0.12 (-0.64, 0.40) | -0.43 (-0.80, -0.06) | -0.21 (-0.55, 0.12) | -0.40 (-0.79, 0.00) | 0.07 (-0.59, 0.73) |
| Diabetes | -0.20 (-0.55, 0.15) | -0.25 (-0.90, 0.39) | 0.49 (-0.09, 1.06) | -0.79 (-1.30, -0.29) | -0.18 (-0.54, 0.19) | -0.40 (-0.90, 0.10) | -0.54 (-1.14, 0.06) |

Note: Estimates (95% CI) are fully adjusted marginal population-averaged coefficients from weighted generalized estimating equation models. Models adjust for all listed variables and survey wave, not shown. Analyses account for the HRS sampling design, survey weights, longitudinal inverse probability weights, and repeated observations within respondents.
